# CYP2E1 inhibition and NF_κB Signaling Pathway are Involved in the Protective Molecular Effect of *Origanum floribundum* against Acetaminophen-induced acute Hepatotoxicity in Rats

**DOI:** 10.22037/ijpr.2021.114487.14878

**Published:** 2021

**Authors:** Amina Khelfallah, Bakhta Aouay, Mohamed Kebieche, Hamadi Fetoui

**Affiliations:** a *Laboratory of Cellular and Molecular Biology, University of Mohamed Seddik Ben Yahia, Jijel, Algeria. *; b *Institute of Veterinary Sciences El-Khroub, University of Constantine 1, Algeria. *; c *Laboratory of Toxicology and Environmental Health, UR11ES70, Sciences Faculty of Sfax, University of Sfax, BP 1171, 3000 Sfax, Tunisia. *; d *Natural and Life Sciences, University of Batna 2. Fesdis, 05000, Batna, Algeria. *

**Keywords:** Origanum floribundum, acetaminophen, oxidative stress, CYP2E1, NF_κB

## Abstract

The present study aimed to estimate the potential and the molecular mechanism of the hydro-ethanolic extract of *O.floribundum* against acetaminophen (AC) induced hepatotoxicity. Four groups of female Wistar rats (n=6) was formed to study the hepatoprotective effect of *O.floribundum* extract against acetaminophen overdose (2 g/kg): Groups N and AC received orally tap water for 03 days and Groups *O. floribundum* + AC and N+*O.floribundum*: received orally* O. floribundum* extract (400 mg/kg). After 1hour (h) of the last dose administered, the paracetamol solution (2 g/kg) is administered orally for group AC and *O. floribundum* + AC. The hydroethanolic extract of *O. floribundum* shows strong antioxidant activity “*in-vitro*”. After 24 h, a single dose of acetaminophen increased significantly serum aspartate aminotransferase (AST), alanine aminotransferase (ALT) and the activity of alkaline phosphatase (ALP) significantly and decreased total protein and albumin levels compared to the normal group. These alterations are confirmed by histological observations with inflammation markers (congestion, inflammatory cells infiltration). These observed effects are mainly due to the over-expression of the *CYP2E1* and *NF_κB* genes marked in this study by quantitative RT-PCR. Also, acetaminophen overdose leads to activation of the mitochondrial permeability transition (MPT). leading to hepatocyte necrosis. Pretreatment with *O.floribundum* before acetaminophen administration removes all previously observed biochemical, histological. and mitochondrial manifestations. These results suggest that *O.floribundum* has a potent antioxidant power and an interesting hepatoprotective activity against acetaminophen toxicity partly due to the inhibition of *CYP2E1* and *NF_κB* genes expression.

## Introduction

The liver is the main organ of drug metabolism, making it the most affected by xenobiotics and often the target of toxicity. Acetaminophen (AC) (xenobiotic) is the most prescribed and popular analgesic and antipyretic in the world. At therapeutic doses, AC is safe. However, after an overdose of AC, detoxification pathways may become saturated, transforming the metabolism of AC to the P450 cytochrome pathway mainly *CYP2E1*, thereby accelerating the generation of *N*-acetyl-*p*-benzoquinone imine (NAPQI) (1). NAPQI is reactive electrophilic and excess NAPQI leads to the depletion of cellular stores of glutathione (GSH) in the liver (2). NAPQI become available and covalently adducts cellular proteins, in particular at the mitochondrial level. Indeed, NAPQI binds to thiol groups in mitochondrial respiratory complexes and exhaust mitochondrial GSH, resulting in an elevation of reactive oxygen species (ROS) and a severe decrease in adenosine-5’ triphosphate (ATP). Therefore, causes hepatocyte death (3).

 AC induced hepatotoxicity promote oxidative stress and redox changes can be important factor in the modulation of signaling pathways for hepatocyte survival or death. The NF_κB signaling pathway is one of the pathways mediating oxidative stress responses and inflammation. Under a redox imbalance and in the absence of an appropriate compensatory reaction of the internal antioxidant systems, the NF_κB pathway was activated (4). NF_κB signaling pathway activation in AC overdose caused the transcription of several inflammation associated genes: *tumor necrosis alpha* (*TNF-α*), *interleukin 1 *(*IL-1*) (5). Indeed, several studies observed that inflammatory reactions might have roles in the establishment of AC-induced liver injury (6). 

Currently, treatments for AC overdose are very restricted. The only antidote proven with antioxidant effect against AC poisoning is N-Acetyl cysteine (NAC) which enhances GSH level and detoxifying NAPQI (7).

The genus *Origanum* L. is an important member of the *Lamiaceae* family. It includes about 43 species (8) which three are found in Algeria: *Origanum glandulosum* Desf. Algerian-Tunisian endemic plant, *Origanum floribundum* Munby (*Origanum cinnereum *de Noe) an endemic and rare Algerian species”Trigger species” and *Origanum majorana* (L.) (9) all commonly known as”Zaatar”.* O. floribundum* is used against diarrhea and others digestives disorders (10). Several pharmacological studies have been carried out on essential oils and phenolic extracts from species of this genus: antioxidant and anti-bacterial activity (11, 12), hepatoprotective (13), anti-inflammatory and cytotoxic (13), anti-diabetic (14, 15). However, based on our knowledge and consulted literature, the reports on the pharmacological properties of *O.floribundum* have not been investigated enough, which may be due to its endemic nature to Algeria.

The purpose of this present research paper was to investigate the hepatoprotective effect of the hydro-ethanolic extract of *O.floribundum* and to study their molecular mechanism against the toxicity of acetaminophen.

## Experimental

*Chemicals*

Acetaminophen (Doliprane®) was purchased from Sanovi-Aventis pharmaceutical co. (Saïdal, Algeria). Kits for the determination of liver enzymes and blood albumin and proteins were purchased from Siemens Medical Solution (Algeria, Alger). Bromohydroxyacetophenone (BHA), butylhydroxytoluene (BHT), ascorbic acid and Kits for the determination of antioxidant activities were purchased from Sigma-Aldrich (GmbH, Steinheim, Germany). Reduced glutathione, thiobarbituric acid (TBA), tri-chloroacetic acid (TCA), bovine serum albumin (BSA) were purchased from Sigma Aldrich (st.Louis, MO). All other chemicals and solvents were of analytical grade.

*Materials*

A 96 wells microplate reader (Perkin Elmer, Enspire^®^ Inc.USA), for the antioxidant activity measurements. System detection CFX96 Real-Time PCR Detection System (BioRad). A Labconco^TM^ lyophilization machine associated with a pump ILMVAC GmbH 302104. Automated Advia^®^ Chemistry Siemens (Germany).

*Collection and authentification of plant*

 The aerial part of *O.floribundum* was collected during the flowering period in July (2014) from Taza National Park (T.N.P.) (Jijel, North East of Algeria) (36°35’-36°48’ North latitude and 5°29’-5°40’ West longitude). The material was identified and authentificated by Mr. Ghribi A. Taza N.P. (Algeria).

*Extraction*

After cleaning, leaves and flowers were dried at room temperature and away from light and mechanically milled. To each 1g of plant, 10 ml of hydro-ethanolic solvent at 60% were added and this operation was carried out for 3 successive days. After that, the solvent has been evaporated at low pressure at 50°C (Rotavapor^®^ Büchi B490) in order to concentrate the extract. The latter has been washed with petroleum ether (3 folds) to get rid of lipids, chlorophyll and then lyophilized for 24 hours. 

*Determination of antioxidant activity*

Antioxidant activities were used by free radical scavenging activity by 1, 1-diphenyl-2-picryl-hydrazyl (DPPH) assay (16). The spectrophotometric evaluation of 2, 2’-azino-bis (3-ethylbenzothiazoline-6-sulphonic acid (ABTS^0+^) scavenging activity was used according to (17). CUPRAC (CUPric Reducing Antioxidant Capacity) antioxidant activity was achieved based on Apak’s approach (18). The reducing power activity is determined by the Oyaizu method (19) with a slight modification. Bromohydroxyacetophenone (BHA), butylhydroxytoluene (BHT) and ascorbic acid were used as antioxidant standards for comparison of the activity. The results are reported as a concentration of inhibition of 50% (CI_50_). The sample concentration that gives 50% activity (CI_50_) was determined from the percentage of inhibitory activity curve in relation to the sample concentration. The concentration of the sample with an absorbance of 0, 5 (A_0, 5_) was calculated from the CUPRAC and reducing power curves and decreasing the absorbance relative to the concentration of the sample.

*Animals*

A group of healthy adult female Wistar rats (n = 24) weighing about 182 ± 9 grams was obtained from Pasteur Institute (Algeria). All animals were housed on metallic cages at an ambient temperature 22 ± 2 °C with a 12h light and dark cycle and allowed free access to tap water and to a standard croquette. Before starting the experiment, they were left 06 rats per cage freely for two weeks. All experimental tests were performed in accordance with the international guidelines to the care and use of laboratory animals. 

*Experimental protocol*

The animals were randomly assigned into four groups (n = 6): control group (tap water for 3 days without AC), AC group (2 g/kg on 3th day), *O.floribundum *+ AC group (400mg/kg of extract of *O. floribundum* for 3 days) and AC (2 g/kg on 3th day), and normal pretreated group (N+ *O.floribundum*) (400 mg/kg of extract of *O. floribundum* for 3 days). Origanum extracts were dissolved in distilled water to give a dose of 400mg/kg. A single dose of AC (2g/kg dissolved in distilled water) was administered orally to AC and (*O.floribundum* + AC) groups 1 h after the last dose of origanum pretreatment on the third day. The dose of AC was determined based on the studies of (20, 21). Rats have fasted overnight, and blood samples were collected from the retro-orbital plexus. After blood collection, all animals were euthanized by ether and thus sacrificed. Liver tissues were then collected rapidly. 

*Biochemical studies*

Blood samples were centrifuged at 3000 rpm for 10 min. Serum aspartate aminotransferase (AST), alanine aminotransferase (ALT) and the activity of alkaline phosphatase (ALP), albumin and proteins levels were determined by automated systems (Advia^®^ Chemistry Siemens, Germany). According to the manufacturer’s instructions, measurements were performed and analyzed using pure kits (Siemens Medical Solutions Diagnostics). 

*Histopathological examination*

Liver tissues were fixed in formalin (10%) and embedded in paraffin and then were sectioned (5 μm) by using a microtome (LEICA RM2235).These sections were colored using hematoxylin and eosin (H.E.). For histological alterations these slides were examined under an optical microscope (LEICA DM 1000.LED).

*RNA extraction and quantitative real-time PCR analysis*

Total RNA was extracted from the frozen liver tissues with Trizol reagent (Bio Basic Inc.) according to the company’s recommendations. RNA levels and purity were quantified by measuring the absorbance A260/280 ratios using a NanoPhotomer^TM ^(Implen, GmBH, Germany). The complementary DNA (cDNA) was synthesized by revere transcription with PrimeScript reverse transcriptase (TaKaRa) as defined in the manufecturer’s instructions using oligo (dT) primer. The real time PCR assay was performed in a total volume of 25µl containing 2µl cDNA, 0,5µl of of each sense and antisense primers (at 10µM): *GAPDH* (forward 5’-AACGACCCCTTCATTGAC-3’and reverse 5’TCCACGACATACTCAGCAC-3’), *CYP2E1* (forward 5’-CTCCTCGTCATATCCATC-3’ and reverse 5’-GCAGCCAATCAGAAATTGG-3’) and *NF_*_K_*B* (forward 5’- GCC GTG GAG TAC GAC AAC ATC-3’ and reverse 5’-TTT GAG AAG AGC TGC CAG CC-3’), and 12,5 µL of 2xPCR Mix (SYBR Premix Ex Taq, TaKara). PCR conditions were set at initial denaturation 95 °C for 3 min followed by 40 cycles consisting of denaturation at 95 °C for 30 s, annealing of primers at 53 °C for 30 s, extinction at 72 °C for 1 min. The ^2−ΔΔ^CT approach was applied to determine the relative expression status of each gene, and the *GAPDH* gene was used as a reference.

*Glycogen content*

 Liver glycogen was measured using Lugol reagent following the procedure described by (22).

*Tissue homogenate preparation*

 Succinctly, rat liver was washed with ice‐cold 0.9% NaCl, plunged in frozen isolation buffer containing TSE (Tris-HCl (50 mM), Sucrose (250mM), and Ethylene diamine tetra acetic acid (EDTA, 1 mM; pH7, 4), and cleared from surrounding tissue and blood. The Liver (about 1 g) was homogenized using an electric homogenizer in 3 volumes of ice-cold TSE, pH7, 4. The crude homogenates were centrifuged twice for 10 min at 800 ×g and 4 °C for eliminating cell debris and nuclei. From the supernatant, liver mitochondria were granulated by centrifugation at 8000 ×g and 4 °C for 10 min. Supernatants from the two centrifugations were mixed and considered as a cytosolic fraction and maintained at -20 °C until the further determination of catalase (CAT), Glutathione S- transferase (GST) activities, GSH and malondialdehyde (MDA) content. While the resultant pellet was washed with TS buffer (50mM Tris-HCL, 250 mM sucrose) pH 7, 4 and suspended in 300µl of TS buffer and frozen at -20 °C until it further uses (23). The mitochondrial suspension was prepared from mitochondria by congelation and decongelation with continuous monogenesis to rupture the membranes of the mitochondria. In order to remove membrane debris, centrifugation at 10000×g for 10 min was done; the supernatant was taken as a supplier of mitochondria GSH, CAT and MDA.

*Redox status evaluation in liver*

To find out the antioxidant effect of the extract in liver with or without AC administration, the following parameters were analyzed: Protein content was established by the Bradford procedure (24) using bovine serum albumin as standard. The activities of CAT and GST were determined using the processes explained by (25) and (26) respectively. Finally, GSH levels were estimated based on Ellman’s test (27) and MDA amounts were measured using the Ohkawa procedure (28).

*Mitochondria swelling essay*

The evaluation of mitochondrial swelling was performed using the Farhi method (29). Same volumes of freshly prepared mitochondria from the liver at 4 ^°^C were read by the optical spectrophotometer at 540nm. The decrease in absorbance indicates the increase in mitochondrial swelling, as a result of the lost potential of the mitochondrial membrane and the mitochondrial opening of the transitional pores of permeability (PTP). 

*Statistical analysis*

Results are expressed as mean value ± standard error meaning (SEM). All statistical comparisons were made by using one-way analysis of variance (ANOVA) followed by Tukey’s multiple comparison tests. Statistical analyses were performed by comparing treatment groups with the normal group (*) and with AC group (#) using GraphPad Prism software (version 6.0 GraphPad Software, Inc., La Jolla, CA, USA). The difference was considered as statistically significant when *P *< 0.05.

## Results

*O. floribundum has a potent antioxidant power “in-vitro”*

The antioxidant activities of hydroethanolic extract of *O. floribundum* were summarized in Table 1. DPPH, ABTS°+ scavenging, CUPRAC and reducing power assays were used to determine the antioxidant activity. BHT and BHA, and Ascorbic acid were the positive standards for comparison of the activity. According to the results observed in Table 1, the *O. floribundum* extract has promising antioxidant activity, since the value of IC_50 _˂ 50 µg/mL in the various tests studied. Consequently, the hydroethanolic extract of *O. floribundum* proved to have antioxidant activity. Furthermore, sometimes the extract demonstrated better activity than the antioxidant standard. In the DPPH and ABTS^°+^ assays, the extract has an IC_50_ equal 15, 69 ± 1 and 7, 15 ± 0, 37 µg/mL respectively. In CUPRAC assay, the *O. floribundum* extract (18, 41 ± 0, 81 µg/mL) is weak only two times than BHT (9, 62 ± 0, 87 µg/mL). Also, in reducing power assay, it has been found that the *O. floribundum* extract (16, 64 ± 0, 9 µg/mL) is only weak about twice as compared to the standard antioxidant BHA (8, 41 ± 0, 67 µg/mL). Furthermore, the reducing power of *O. floribundum* is better than BHT antioxidant standard (A_0, 50_ ˃ 50 µg/mL). 

*O.floribundum suppresses paracetamol-induced hepatotoxicity in rats*

AC treatment significantly increased the activity of the hepatic transaminase enzymes in serum: AST, ALT and ALP. Nevertheless, a significant decrease in total protein and albumin levels was also noted. These results confirm the hepatotoxic effect of acetaminophen at 2 g/kg. Animals that received oral doses of *O.floribundum* extract plant (400 mg/kg body weight) for 3 days before AC treatment indicates marked enhancement of the most markers of biochemistry compared to normal levels of untreated controls (Table 2). 

*Hepatic histopathological examination*

**Figure 1:**
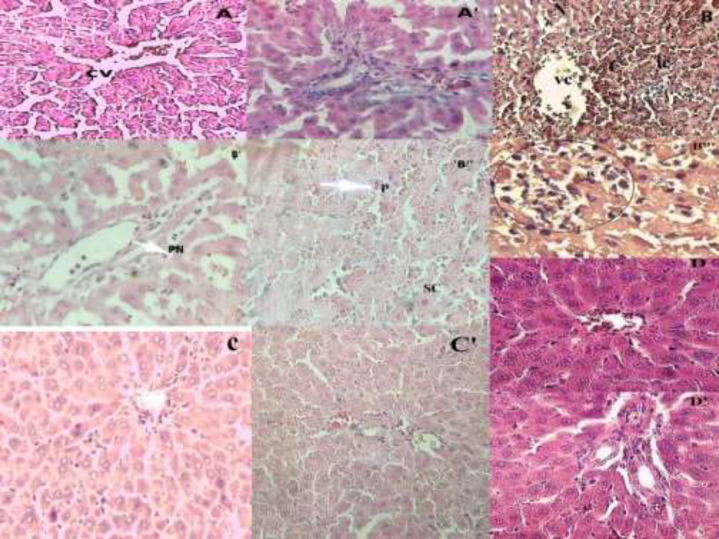
Effect of *O. floribundum *extract on liver histological changes following AC toxicity in rats: (A) control group showing the normal structure of the liver, (A') normal portal space of control group with veins and arteries of regular structure; (B, B", B'"): AC treated groups with massive Porto-centrilobular necrosis. Swollen hepatocytes (B") hepatocyte with nuclear pyknosis (B") inflammatory cells infiltration for (B'’’), B’: portal space of AC treated group with piecemeal necrosis, Figure C: *O. floribundum *+AC pretreated group showing sub-normal architecture, Figure C’: normal portal space of *O floribundum*+AC pretreated group Figure D: normal liver for *O. floribundum *pretreated group, Figure D’: normal portal space of *O. floribundum *group (Hematoxylin Eosin X200); X400 for figure B”’. CV: centrilobulare vein; C: congestion; P: pyknos; PN: pieacemeal necrosis; N: necrosis; SC: swelling cell, IC: inflammatory cells

Figure 1A demonstrates a control rat liver area showing the ordinary histological structure of the hepatocytes and sinusoidal space. The portal space (Figure 1A’) is also normal with veins and arteries of regular structure. However, when contrasted with liver histological microphotograph from the control group (Figure A), liver segments of the AC-treated group (Figure 1 B, B’, B”) displayed serious disarrangement of hepatic cells with massive Porto-centri-lobular necrosis. Swollen hepatocytes (Figure 1B”) were observed that are in the process of necrosis and nuclear pyknosis (Figure 1B”). Also, the hepatic parenchyma presents dilated sinusoids with inflammatory cells infiltration (Figure 1B”) and vascular congestion. On the other hand, the portal space of the AC-treated group (Figure 1B’) with piecemeal necrosis without fibrosis and septa, congestive vessels with a turgescence of endothelial cells and inflammatory cells infiltration. Figure 1C shows the liver section of an *O.floribundum* pretreated (400 mg/kg) rats that also received AC strangely remove completely the histopathological variations inflammatory changes related to AC overdose as well as in portal space (Figure 1C’). Figure 1D shows the liver section of rats pretreated alone with *O.floribundum* (400mg/kg) with normal hepatocytes nuclei and normal sinusoidal space. The portal space of the normal group pre-treated with *O.floribundum *is also intact (Figure 1D’). The results of the semi-quantitative analysis are reported in Table 3.

*O.floribundum inhibited AC induced oxidative stress injury and inflammatory responses in rat*

**Figure 2:**
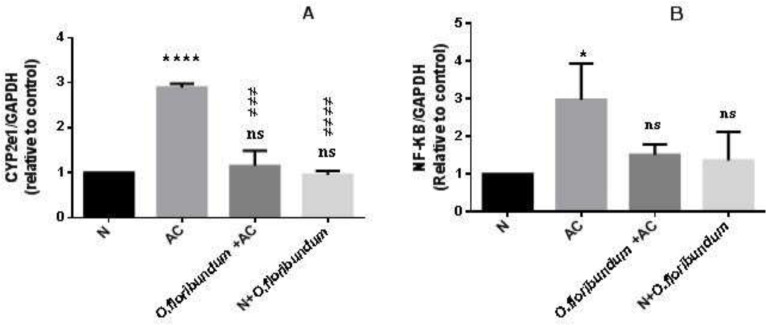
(A and B) Study of the *CYP2E1* and *NF-*_K_*B* genes expression by qRT-PCR in the liver rat

Currently, it is known that the metabolism of AC in the liver is mainly under the action of CYP2E1. To see if *O.floribundum* has an effect on the expression of the *CYP2E1 *gene, qRT-PCR was performed. Paracetamol treatment significantly increased* CYP2E1* gene expression compared to the normal group (*P* ˂ 0.0001). However, pretreatments with *O.floribundum *normalized *CYP2E1* gene expression significantly compared to the AC group (*P* ˂ 0.0001). Treatment with *O.floribundum* does not change *CYP2E1* gene expression compared to the normal group (Figure 2 A). 

The NF__K_B signaling pathway is known to regulate the expression of various genes involved in the inflammatory response. To analyze the effect of *O.floribundum* on *NF_*_K_*B* gene expression, qRT-PCR was carried out. The expression of the *NF_*_K_*B* gene in the AC group was elevated significantly compared to the normal group (*P* ˂ 0.05).Whereas pretreatment with *O.floribundum* (400 mg/kg) reversed the elevation of NF__K_B gene expression and it is not significantly different from the normal group. Pretreatment with *O.floribundum* alone does not change the expression of the *NF_*_K_*B* gene compared to the normal group (Figure 2B).

*Effect of O.floribundum on liver glycogen content*

**Figure 3:**
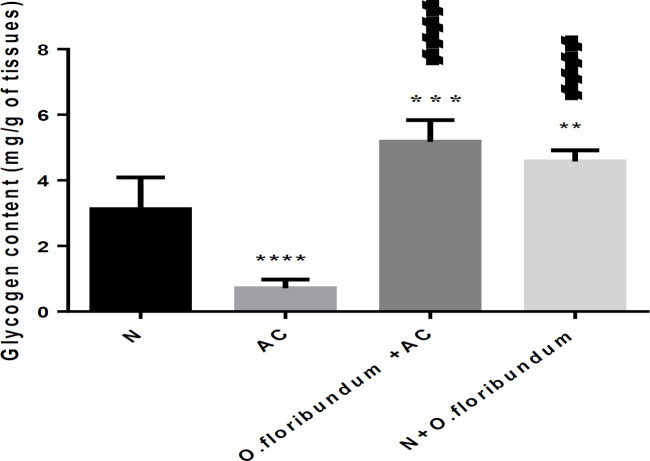
effect of *O. floribundum* on liver glycogen content in different treated groups

A single dose of AC treatment (2 g/kg) decreases the concentration of hepatic glycogen very significantly by about 77% compared to the normal group (*P* ˂ 0.0001), whereas pretreatment with *O.floribundum* enhances significantly glycogen level by about 66% compared to the normal group (*P* ˂ 0.01). Pretreatment with *O.floribundum* alone significantly enhances the level of glycogen by about 47% compared to the normal group (Figure 3).

*Effect of O.floribundum on oxidative stress markers both in cytosolic and mitochondrial liver intoxicated rats*

*Effect of O. floribundum on cytosolic and mitochondrial GSH content*

 Hepatic GSH contents at cytosolic and mitochondrial levels in different groups are shown in Table 4. AC caused a significant depletion in GSH levels in the cytosol (by 35%) and mitochondria (33%) of liver homogenates compared to the normal group (*P* ˂ 0.05 and *P* ˂ 0.0001) respectively. Pretreatment with *O. floribundum* enhance GSH level significantly in the cytosol and mitochondria of liver homogenates by 100% and 42% respectively compared to AC group. In addition, the cytosolic GSH level obtained from rats pretreated with *O. floribundum* with or without AC administration is significantly elevated compared to the normal group (*P* ˂ 0. 01 and *P* ˂ 0.05) respectively. Whereas, this effect is not significantly different for GSH level in mitochondria liver homogenate compared to normal group both after administration of paracetamol or not (Table 4).

*Effect of O. floribundum on cytosolic and mitochondrial rat liver lipid peroxidation*

Lipid peroxidations on cytosolic and mitochondrial liver homogenates were estimated by measuring thiobarbituric acid reactive substances (TBARS) as shown in Table 4. Paracetamol elevated MDA levels in the cytosol and mitochondria of liver homogenates significantly, respectively three to almost two times compared to the normal group. Pretreatment with *O. floribundum* ameliorated this effect by nearly (47%) in cytosolic and (45%) in mitochondrial liver homogenates compared to the AC group. Pretreatment with *O. floribundum* alone has not changed MDA amounts in cytosolic and mitochondrial liver homogenates compared to the normal group (Table 4).

*Effect of O. floribundum on cytosolic and mitochondrial activities of catalase and glutathione S-transferase antioxidant enzymes*

Compared to the normal control group, homogenate of the liver from the AC group proved a significant decrease (*P* ˂ 0.01 and *P* ˂ 0.05) in CAT activities at the cytosolic and mitochondrial level (-76% and -53%) respectively. Pre-treatment with *O.* *floribundum* at a dose of 400mg/kg significantly increased CAT activity (*P* ˂ 0.01) at the cytosolic level compared to the toxic control group, to a level close to that of the normal control group (Table 5). However, the mitochondrial CAT activity of the *O.floribundum*+AC group increased but remains not significantly different compared to the intoxicated control group.

Similarly, GST activities in cytosolic liver homogenate from the AC group are also decreased significantly (*P* ˂ 0.01) compared to the normal control group. Pre-treatment with *O. floribundum* before administration of AC was significantly different (*P* ˂ 0.05) compared to AC intoxicated control group, to a level close to that of the normal group (Table 5).

Administration of *O. floribundum *alone does not have an impact on the activities of CAT and GST at cytosolic and mitochondrial levels and the values are not significantly different compared to the normal group (Table 5). 

*Effect of O. floribundum on mitochondrial swelling*

In previous studies, mitochondrial dysfunction has been reported in an AC overdose. In order to confirm the possible preventive effect of *O. floribundum *extract, the optical densities at 540 nm of liver mitochondrial suspensions extracted freshly were recorded in *O.floribundum* and/or AC treated groups.

Compared to the normal control group, the optical density of liver mitochondrial suspensions of the AC**_**treated group decreased very significantly (P ˂ 0.001) (-35%). Whereas pre-treatment with *O. floribundum *before administration of AC improved the value by 18% compared to the intoxicated control group. The normal group treated with *O.floribundum *alone has the optical density (1, 42 ± 0, 23) not significantly different from the normal control group (Table 6).

## Discussion

This study was carried out for the first time on a rare and endemic species of Algerian flora: *Origanum floribundum *(Lamiaceae family). Moreover, pharmacological studies on this species are very limited. We are trying to evaluate the hepatoprotective effect of lyophilized hydro-ethanolic extract of *O. floribundum *and to understand its molecular mechanisms on an acute model of hepatoxicity induced by a single toxic dose of paracetamol (2 g/kg). 

 Currently, scientists have a strong interest in naturally occurring secondary metabolites. They have examined their antioxidant properties that may be the treatment of various ailments due to their health benefits (30). In terms of their functions, the antioxidants can be classified into scavenger antioxidants, preventive antioxidants and *de novo* and repair antioxidants (31). Consequently, the antioxidant activity of *O. floribundum* extract was carried out using four different assays under different conditions: the capacity of hydroethanolic extract for scavenging free radicals was assessed by two methods: DPPH free radical scavenging and ABTS^°+^ cation radical discoloration. Assessment by reduction of cupric (Cu II) to their lower valiancy state known as the CUPRAC method was used. Finally, the reducing power assay was carried out. 

The effect of antioxidants on DPPH or ABTS radicals scavenging is achieved by hydrogen or electron transfer followed by proton transfer to become stable molecules (31). The reaction of antioxidants with DPPH or ABTS^°+^ is followed by a decrease in their absorption at 517 and 734nm respectively. The relative antioxidant capacity for scavenging radicals is expressed by inhibitory concentration (IC_50_), which corresponds to the concentration of the extract to obtain a 50% reduction in the initial free radical (DPPH or ABTS) concentration. A higher radical capture activity is associated with lower IC_50_ levels. Following our results, the hydro-ethanolic extract of *O. floribundum* has a potent antioxidant. These results are related to the high content of phenolic compounds. Also, it is known that phenolic compounds act mainly as (primary) scavenger antioxidants of free radicals. 

In the literature, a powerful radical scavenging antioxidant usually functions as a powerful reducer (31). The CUPRAC method is based on the reaction between complexes of probes (Nc) with the reduced form of the metals giving visible absorption bands with maximum intensities at 450nm (32). The reductive potential is associated with antioxidant activity. The reducing power property of an extract indicates the capacity to act as an electron donor by which the lipid peroxidation process can be stopped by reducing oxidative products to progressively stable substances (33). In this study, the *O. floribundum* extract gave an absorbance value 02 folds higher than that expressed by BHA standard (A_0, 5 _values: 16, 64 ± 0, 9 and 8, 41 ± 0, 67 µg/ml respectively) that means, the reducing power of the standard antioxidant is more than twice as high as that of the extract tested. Therefore, the extract has a powerful reducing power.

AC induced liver injury and has served as the most important model, useful for testing the hepatoprotective activity of medicinal plants (34). After 24 h of AC intoxicated rats, abnormal liver indicators become apparent in serum include by elevated serum aminotransferases activities (AST, ALT) and ALP. Since these enzymes are exclusively intracellular, their release is explained by the loss of integrity of the hepatocytes’ membranes and lipids’ peroxidations (35, 36). These findings are confirmed after the observation of histological sections that demonstrate massive necrosis caused by AC. Furthermore, significant reductions in serum protein and albumin levels were marked. These results are related to the loss of functional integrity of the liver and a reduction in the number of hepatocytes through necrosis (30, 37). The animals pre-treated with *O. floribundum *(400 m/kg) revealed significant protection of liver tissue against AC that appeared in the normalization of serum levels of liver function markers (Table 2) and was confirmed after observation of histological sections (Figure 1). These results concluded that *O. floribundum *has a promising hepatoprotective effect.

AC-induced hepatotoxicity is conducted by the action of cytochrome P 450 mainly CYP2E1 to form reactive metabolites: N-acetyl p-benzoquinone (NAPQI) and releases a significant amount of ROS. High levels of NAPQI induce a decline in the GSH stores in cytosolic and mitochondrial levels (5). The decrease in mitochondrial GSH levels is explained by the presence of CYP2E1 also into mitochondria (38) and in this way, NAPQI is generated within to mitochondria. GSH depletion is the main event that initiates lipid peroxidation and protein adducts formation especially in the mitochondrial respiratory chain. Consequently, it promotes overproduction of ROS and selective oxidant stress. This aggravates paracetamol-induced hepatotoxicity.

Since cytochrome P450 particularly CYP2E1, is the main oxidation system of paracetamol into NAPQI and ROS that triggers a cascade of the process resulting in hepatotoxicity. Also, the overexpression of the cytochorome P450 *CYP2E1*gene in AC overdose is confirmed by several studies (39, 40). We investigated the effect of *O.* *floribundum *extract on the expression of the *CYP2E1* gene. As expected, in the present study, AC overdose induces *CYP2E1* gene expression very significantly. However, pre-treatments with *O. floribundum *extract normalize *CYP2E1* gene expression to a level similar to the normal group. Interestingly, this finding supported for the first time that *O.* *floribundum* has a molecular protection mechanism against AC through downregulation of *CYP2E1* gene expression. This result means that *O.floribundum *extract suppresses the conversion of AC into NAPQI and free radicals in the liver and thereby could explain the improvement of GSH level and redox status. Therefore, one of the molecular mechanisms of the protection of *O. floribundum* against paracetamol-induced hepatotoxicity is interaction with the expression of *CYP2E1* gene.

Several previous reports exhibit that polyphenols may be effective in regulating the cytochrome CYP450 system via the inhibiting of their activity, the decline in their liver content and their expression (41). For example, rosmarinic acid caused marked inhibition of the CYP2E1 enzymatic activity in AC overdose (42). This phenolic acid is an excellent chemotaxonomic marker present in the Nepetoideae subfamily where our study plant belongs: *Origanum floribundum*. However, according to the literature review, this phenolic acid is not yet confirmed in the phenolic composition of *O. floribundum. *

NF_kB is a transcriptional factor related to stress-induced inflammation (43). In this study, we found several markers of inflammation at the tissue level in the AC intoxicated group, such as severe congestion, sinusoid dilation, and inflammatory cell infiltration. These results are in agreement with several previous studies, which often linked inflammatory response with AC-induced liver damage (5, 44). However, treatment with *O. floribundum *extract declines all these events. The above data indicate that activation of the NF_κB signaling pathway is linked to inflammatory response and cellular dysfunction and damage (43). In the present study, AC intoxication resulted in over-expression of the *NF_κB* gene, which is in concurrence with (45), who detected strong phosphorylated protein expressions of NF_κB in AC overdose. Interestingly, in the present work, we demonstrated for the first time that *O. floribundum *reduced *NF_κB* gene expression-induced hepatic inflammation in rats. In a previous study, *Origanum majorana* extract was revealed to suppress *NF_κB* activation against breast cancer via anti-metastatic activity (46). Similarly, this study demonstrated that *O.floribundum* significantly attenuated liver injury in rat partly via the inhibition of *NF_κB *gene expression.NF_κB signaling pathway activation in AC overdose caused the transcription of several inflammation-associated genes (*TNF-α, IL-1B,IL- 6*) (5, 43). It, therefore, seems that the inhibitory effect on the expression of the *NF_κB *gene may be the result of the anti-inflammatory effects of *O. floribundum*. 

Among the specific functions of the liver is the storage of glycogen. In this study, AC intoxication resulted in depletion of liver glycogen which is in concurrence with many research (47, 48). Indeed, in the case of paracetamol intoxication, several authors confirm the activation of glycogen degradation via the glycogenolysis pathway (49) by activating the key enzyme of glycogenolysis: glycogen phosphorylase A (50). In fact, the main pathways of AC metabolism are conjugation through glucuronosyl-transferase and sulfo-transferase to produce no toxic metabolites. These routes depend on the accessibility of their co-substrates: UDP-glucuronic acid (UDPGA) and 3’-phosphoadenosine5’-phosphate (PAPS), respectively. The latter is provided by glycogenolysis (51). On the other hand, the glycogen level in the animals intoxicated and pre-treated by *O. floribundum *is significantly high compared to the normal group. We suggest, in this case by the effect of the bio-molecules present in the extract of the plant to increase a certain growth under the effect of the toxic and resist the damage caused by AC through prevention of glycogenolysis. 

From the results found above, it can be explained that paracetamol overdose increased oxidative stress and mitochondrial dysfunction. Indeed, depletion of GSH and elevation of MDA end products both in cytosolic and mitochondrial levels were recorded. Moreover, we registered a decrease in catalase activities both in cytosol and mitochondria and glutathione S-transferase activities in the cytosol. 

The protective effect of *O.floribundum* could be associated with its ability to prevent the increase of oxidative damage and decrease of antioxidant enzyme activity. Interestingly, we have found that *O. floribundum* increased the normal level of cytosolic GSH significantly. Well, these results are partly due to the high contents of *O.floribundum *in phenolic and flavonoïds compounds, secondary metabolites known for their antioxidant power. This later has been proven in this study by several different tests “*in vitro*”. In addition, there are some studies on the effects of particular flavonoids on glutathione levels. Moskaug has been found that quercetin increases the expression of γ-glutamylcysteine synthetase with a concomitant increase in the intracellular glutathione (52). A preliminary analysis (Result not yet published) by chromatographic separation coupled with mass spectrometry (GC-MS) shows that the extract of *O. floribundum* is very rich in bioactive molecules (52 compounds), including fatty acids (n-Hexadecanoic acid), catechol and, indole. A derivative of indole has been proven by Park (53) to be able to combine directly with NAPQI. According to these results and for the first time, *O.floribundum* treatment can increase GSH content and induce both enzymatic and no enzymatic antioxidant capacity. 

Mitochondrion plays a key function in cells survival. Indeed, it is responsible for 95% of cellular energy needs. In this study, we recorded mitochondrial dysfunction through depletion of glycogen, stress oxidative and, mitochondrial swelling. On the other hand, *O.floribundum *pretreatment demonstrates a protective effect on AC-induced mitochondrial dysfunction. It is well established that NAPQI overproduction inset the mitochondria block the respiratory chain, increases cell calcium, the formation of ROS including peroxynitrite and, activation of the mitochondrial permeability transition leading to hepatocyte necrosis. The protective effect of *O. floribundum *on AC-induced mitochondrial dysfunction could be associated with its antioxidant effects.

N: normal control group received tap water, AC: acetaminophen intoxicated group (2 g/kg on 3th day), *O. floribundum*+AC: *O. floribundum *pretreated group (received 400 mg/kg of *O. floribundum* extract for 3 days then 2 g/kg of AC at 3th day. N+*O. floribundum*: normal pretreated group with O. floribundum for 3 days. All values are expressed as means ± SEM; n = 6. One-way ANOVA followed by Tukey test. ns : not significatif (*p* ˃ 0,05) ;(^*^p ˂ 0,05) ; (^****^) *p* ˂ 0,0001 towards the normal group (^###^) *p* ˂ 0, 001; (^####^) p ˂ 0, 0001 towards the intoxicated group (AC).

N: normal control group received tap water, AC: acetaminophen intoxicated group (2 g/kg on 3th day), O+AC: *O. floribundum* pretreated group (received 400 mg/kg of *O. floribundum* extract for 3 days then 2 g/kg of AC at 3th day. N+O: normal pretreated group with *O. floribundum* for 3 days. All values are expressed as means ± SEM; n = 6. One-way ANOVA followed by Tukey test.(^*^p ˂ 0,05) ; (^***^) *p* ˂ 0,001 towards the normal group. (^#^) *p* ˂ 0, 05 towards the intoxicated group (AC). (^**^) *p* ˂ 0, 01; (^***^) *p* ˂ 0, 001; (^****^) *p* ˂ 0, 0001 towards the normal group. (^####^) *p* ˂ 0, 0001 towards the intoxicated group (AC).

**Table 1:** Antioxidant activity of *Origanum floribundum* hydro-ethanolic extract by DPPH_, ABTS_+, CUPRAC and reducing power assays

**Extract**	**Antioxidant activity**			
	**DPPH assay**	**ABTS assay**	**CUPRAC assay**	**Reducing power assay**
	**CI**_50_ ** (µg/mL)**	**CI**_50_ ** (µg/mL)**	**A**_0,5 _ **(µg/mL)**	**A**_0.5 _ **(µg/mL)**
Extract of *O.* *floribundum*	15,69* ± *1,00	7,15* ± *0, 37	18, 41* ± *0, 81	16, 64* ± *0, 9
BHT^a^	22, 32* ± *1, 19	1,59* ± *0,03	9, 62* ± *0, 87	>50
BHA^a^	5, 73* ± *0, 41	1,03* ± *0,0	3, 64* ± *0,19	8, 41* ± *0, 67
Ascorbic Acid^a^	Nt	Nt	Nt	9, 01* ± *1, 46

(CI_50_): concentration inhibition 50% and (A_0, 5_): Absorbances _0, 5_ values expressed are means ± SEM of three parallel measurements (*p* ˂ 0, 05).

^a^antioxidant standard, BHT: butylhydoxytoluene; BHA: Bromohydroxyacetophenone; Nt: not tested; DPPH: 1, 1-diphenyl-2-picryl-hydrazil;

ABTS_^+^: 2, 2'-azino-bis (3-ethylbenzothiazoline-6-sulphonic acid); CUPRAC: CUPric Reducing Antioxidant Capacity.

**Table 2:** The effect of *O. floribundum *hydro-ethanolique extract on biomarker of hepatic damage

**Groups**	**ALT** **(IU/L)**	**AST** **(IU/L)**	**ALP** **(IU/L)**	**Albumin** **(g/L)**	**Protein** **(g/L)**
N	68,21 *± *17,53	180,24 *± *51,75	119,33 *± *28, 44	40,38 *± *1,25	78,93 *± *2,74
AC	1384,13* ± *197,58^****^	2636,71* ± *243,69^****^	200,66* ± *62,36^*^	37,19* ± *0, 46^*^	73,08 *± *1,46^**^
O+AC	72,84 *± *11,55^ns^^####^	236,22* ± *30,82^ns####^	113,83* ± *42,25^ns#^	39,69* ± *1,28^ns^	78,53 *± *3,33^ns##^
N+O	61,87 *± *23,91^ns^^####^	181,98* ± *35,55^ns####^	97,66* ± *29,17^ns##^	39,86* ± *0,75^ns^	78,58* ± *2,92^ns##^

N: normal control group received tap water; AC: acetaminophen intoxicated group (2 g/kg on 3 th day), O+AC: O. floribundum pretreated

group (received 400 mg/kg of O. floribundum extract for 3 days then 2 g/kg of AC at 3th day. N+O: normal pretreated group with

*O. floribundum* for 3 days. AST: Serum aspartate aminotransferase; ALT: alanine aminotransferase and ALP: alkaline phosphatase.

All values are expressed as means ± SEM; n = 6. One-way ANOVA followed by Tukey test. Ns : not significatif (*p* ˃ 0,05) ;(^*^p ˂ 0,05) ;

(^**^) *p *˂ 0,01 ; (^***^) *p *˂ 0,001 ; (^****^) *p *˂ 0,0001 towards the normal group. (^#^) *p* ˃ 0, 05; (^##^) *p *˂ 0, 01; (^###^) *p *˂ 0,001; (^####^) *p *˂ 0, 0001

towards the intoxicated group (AC).

**Table 3:** The results of semi- quantitative hepatic examination

**Groups**	**Necrosis**	**Congestion **	**Inflammatory cells **	**Pyknosis **	**Portal space **
N	**-**	**-**	**-**	**-**	**-**
AC	**++++**	**++++**	**+++**	**+**	**+**
O+AC	**-**	**-**	**+**	**-**	**-**
N+O	**-**	**-**	**-**	**-**	**-**

Note: -: No defects noted, +: injuries/ alterations up to less than 25%; +++: injuries/ alterations up to less than 75%;

++++: injuries/ alterations up to more than 75%. N: normal control group received tap water, AC: acetaminophen

intoxicated group (2 g/kg on 3th day), O+AC: *O. floribundum* pretreated group (received 400 mg/kg of O. floribundum

extract for 3 days then 2 g/kg of AC at 3th day. N+O: normal pretreated group with O. floribundum for 3 days.

**Table 4.** The effect of pre-treatment with *O. floribundum* on cytosolic and mitochondrial GSH and MDA end product content after AC administration

**Groups**	**GSH content **		**MDA content **	
	**Cytosolic Level** **(µmol/mg proteins)**	**Mitochondrial level(µmol/mg proteins)**	**Cytosolic** **Level (µmol/mg proteins)**	**Mitochondrial level(µmol/mg proteins)**
N	0,45* ± *0,04	0,143* ± *0,0	0,566* ± *0,25	0,560* ± *0,11
AC	0,296* ± *0,05^*^	0,096* ± *0,005^****^	2,07* ± *0,65^****^	0,860* ± *0,07^***^
*O* +AC	0,619* ± *0,12^**####^	0,137* ± *0,048^ns^	1,09* ± *0,45^ns##^	0,455* ± *0,06^ns####^
N+*O*	0,584* ± *0,072^*####^	0,162* ± *0,017^ns^	0,830* ± *0,27^ns###^	0,599* ± *0,04^ns##^

N: normal control group received tap water, AC: acetaminophen intoxicated group (2g/kg on 3th day), O+AC: O. floribundum pretreated group

(received 400 mg/kg of O. floribundum extract for 3 days then 2 g/kg of AC at 3th day. N+O: normal pretreated group with *O. floribundum *for 3 days.

GSH: glutathione, MDA: malondialdehyde All values are expressed as means ± SEM; n = 6. One-way ANOVA followed by Tukey test. ns : not

significatif (*p *˃ 0,05) ;(^*^p ˂ 0,05) ; (^**^) *p *˂ 0,01 ; (^***^) *p *˂ 0,001 ; (^****^) *p *˂ 0,0001 towards the normal group. (^#^) *p* ˂ 0, 05; (^##^) *p *˂ 0, 01; (^###^) *p *˂ 0, 001;

(^####^) *p *˂ 0, 0001 towards the intoxicated group (AC).

**Table 5:** The effect of pre-treatment with *O. floribundum* on cytosolic and mitochondrial catalase activity and cytosolic GST activity

**Groups **	**Catalase activity **		**GST activity**
	**Cytosolic Level** **(µmol/mg proteins)**	**Mitochondrial level(µmol/mg proteins)**	**Cytosolic** **Level (IU/mg proteins)**	
N	0,11* ± *0,02	0,0938* ± *0,04	0,101* ± *0,03	
AC	0,026* ± *0,01^**^	0,044* ± *0,021^*^	0,046* ± *0,01^*^	
*O* +AC	0,10* ± *0,05^ns#^	0,0823* ± *0,01^nsns^	0,098* ± *0,029^ns#^	
N+*O*	0,13* ± *0,02^ns###^	0,105* ± *0,01^ns##^	0,118* ± *0,02^ns##^	

N: normal control group received tap water, AC: acetaminophen intoxicated group (2g/kg on 3th day), O+AC: *O. floribundum*

pretreated group (received 400 mg/kg of *O. floribundum* extract for 3 days then 2 g/kg of AC at 3th day. N+O: normal pretreated

group with *O. floribundum* for 3 days. GST: glutathione S- Transferase. All values are expressed as means ± SEM; n = 6.

One-way ANOVA followed by Tukey test. ns : not significatif (*p* ˃ 0,05) ;(^*^) *p* ˂ 0,05) ; (^**^) *p* ˂ 0,01 ; towards the normal group.

(^#^) *p* ˂ 0, 05; (^##^) *p* ˂ 0, 01; (^###^) *p *˂ 0, 001 towards the intoxicated group (AC).

**Table 6.** The effect of *O. floribundum *on the prevention of AC-induced mitochondrial dysfunction by measuring the optical density at 540 nm of mitochondrial suspensions in the liver

**Groups**	**Optical density at 540nm**
N	1,709* ± *0,09
AC	1,104* ± *0,28^***^
*O* +AC	1,307* ± *0,27^*^
N+*O*	1,481* ± *0,11^#^

N: normal control group received tap water, AC: acetaminophen intoxicated group (2 g/kg on 3th day), O+AC:

*O. floribundum* pretreated group (received 400 mg/kg of *O. floribundum* extract for 3 days then 2 g/kg of AC at

3th day. N+O: normal pretreated group with *O. floribundum* for 3 days. All values are expressed as means ± SEM;

n = 6. One-way ANOVA followed by Tukey test. ns : not significatif (*p* ˃ 0,05) ;(^*^p ˂ 0,05) ; (^***^) *p* ˂ 0,001

towards the normal group. (^#^) *p* ˂ 0, 05 towards the intoxicated group (AC).

## Conclusion

From the research that has been carried out, we have proved for the first time that the lyophilized extract of *Origanum floribundum *(Lamiaceae) from Algeria has a significant hepatoprotective power against the acute toxicity of paracetamol. In addition, the species is capable of decreasing the expression of the *CYP2E1 *gene, the main isoform of cytochrome P450 responsible for the oxidation of acetaminophen to reactive metabolite (NAPQI). In addition, *O. floribundum* has an inhibitory effect on the expression of the *NF_κB* gene through its anti-inflammatory action against paracetamol toxicity. Also, the lyophilized extract of *Origanum floribundum *proved its antioxidant power “*in -vitro*” and pretreatment with *O. floribundum* before the acetaminophen intoxication enhance the antioxidant power “*in-vivo*” and decrease oxidative stress simultaneously in the cytoplasm and mitochondria of the liver. In perspective, further studies are required to identify bioactive molecules, particularly their phenolic composition, and to identify those responsible for its observed beneficial effects.

## Ethical statement

All experimental trials were conducted in conformity with international guidelines on the care and use of laboratory animals. 

## Conflict of interests

There is no conflict of interest to be mentioned.

## Study of highlights

*What is the current knowledge?*

High-dose paracetamol is hepatotoxic. It induces over expression of *CYP2E1 *and *NF_*_K_*B* genes. This leads to oxidative stress, inflammation and mitochondrial dysfunction. 

*What is new here?*

This study approves for the first time that *Origanum floribundum *from Algeria (Lamiaceae) has a hepatoprotective effect through the modulation of the expression of the *CYP2E1* and *NF-*_K_*B* genes and exercised an antioxidant power “*in-vivo*”.

## Authors’ contribution 

Amina Khelfallah was responsible for the conception and execution of the experiments. Bakhta Aouey realized and analyzed the data of the molecular part. Hamadi Fetoui provided analytical materials and tools. Amina Khelfallah wrote the paper. Mohammed Kebieche supervised all parts of this study and examined the paper.
